# Patient-reported oral complaints during cancer treatment: Dietary consequences, supportive care, and self-management in a cross-sectional survey

**DOI:** 10.1186/s12903-026-10389-0

**Published:** 2026-11-03

**Authors:** Enno Dosch, Jutta Hübner, Clemens Wolf, Jens Büntzel, Luca Schmidt

**Affiliations:** 1 Klinik für Innere Medizin II, Hämatologie und Internistische Onkologie, Universitätsklinikum Jena, Am Klinikum 1, Jena, 07747 Germany; 2 Comprehensive Cancer Center Central Germany, Campus Jena, Jena, Germany; 3 Klinik für HNO-Erkrankungen, Kopf-Hals-Chirurgie, Südharz-Klinikum Nordhausen, Nordhausen, Germany

**Keywords:** Oral health, Neoplasms, Xerostomia, Dysgeusia, Food intake, Patient reported outcome measures

## Abstract

**Background:**

Oral complaints are common during cancer treatment and may interfere with eating and perceived quality of life. However, patient-reported evidence addressing dental and gingival complaints, xerostomia, taste alterations, counselling, and self-management remains limited.

**Methods:**

We conducted an anonymous cross-sectional survey among adults in Germany with current or previous oncological treatment using paper-based and online questionnaires (January-June 2025). Analyses were based on valid responses for each item and included descriptive statistics, Pearson’s chi-square tests, linear-by-linear association tests, 95% confidence intervals for selected key prevalence estimates, and exploratory multivariable logistic regression sensitivity analyses adjusted for age and gender.

**Results:**

The survey included 222 patients. New, additional, or worsening dental and gingival complaints during cancer treatment were reported by 41.9% (83/198; 95% CI 35.3–48.9%) of respondents. Xerostomia was reported by 56.6% (120/212; 95% CI 49.9–63.1%) and taste alterations by 68.0% (138/203; 95% CI 61.3–74.0%). Self-reported reduced food intake attributed to dental or gingival complaints was reported by 27.9% (57/204; 95% CI 22.2–34.5%), while 25.0% (46/184; 95% CI 19.3–31.7%) reported perceived quality-of-life impairment attributed to these complaints. In exploratory sensitivity analyses adjusted for age and gender, new, additional, or worsening complaints remained associated with reduced food intake (adjusted OR 7.85, 95% CI 3.70-16.62) and perceived quality-of-life impairment (adjusted OR 15.25, 95% CI 5.81–40.06). Among participants with xerostomia, the proportion reporting reduced food intake increased from 5.1% among those with no xerostomia-related eating difficulty to 91.7% among those with severe difficulty (*p* < 0.001). Overall, 43.1% (87/202; 95% CI 36.4–50.0%) reported receiving support or counselling regarding oral health.

**Conclusions:**

In this recruited sample, oral complaints during cancer treatment were common and were associated with eating-related burden and perceived quality-of-life impairment. These exploratory associations persisted after limited adjustment for age and gender but do not establish causality. Fewer than half of respondents reported receiving oral-health counselling, although perceived helpfulness was high among recipients, suggesting a potential gap in the reach of such support. These findings support prospective evaluation of repeated, function-oriented oral-health assessment and appropriate dental or nutritional referral rather than demonstrating the effectiveness of these approaches.

**Supplementary Information:**

The online version contains supplementary material available at https://doi.org/10.1186/s12903-026-10389-0.

## Introduction

Oral discomfort is a clinically relevant and heterogeneous consequence of cancer treatment. Chemotherapy, radiotherapy, targeted therapies, and immunotherapies can affect the oral mucosa, salivary glands, taste perception, teeth, and gingiva, although the type and severity of complications vary according to treatment modality and individual patient factors [1–3]. Patients may experience mucosal soreness or ulceration, xerostomia, dysgeusia, dental or gingival complaints, oral pain, denture-related difficulties, and impaired chewing or swallowing [1, 4]. These problems may arise during treatment and, depending on the treatment modality, persist beyond the active treatment phase [1, 5].

Maintaining oral health is therefore an important component of supportive cancer care. Current clinical practice statements emphasise preventive oral care, early recognition and management of treatment-associated complications, and collaboration between oncology professionals and dental care providers [2, 3, 6]. In Germany, the current S3 guideline on supportive care recommends structured oral-care measures and symptom-oriented management of treatment-related oral complications, including oral mucositis and xerostomia [7]. Medically indicated dental diagnostic and treatment measures are generally available within statutory dental care. However, coverage and potential patient co-payments depend on the specific service, particularly for dental prosthetic treatment [8, 9]. Nevertheless, patient-reported studies and expert assessments suggest that oral and dental care are not consistently integrated into cancer care and that some patients receive insufficient information or support regarding treatment-associated oral problems and appropriate self-care measures [10, 11]. Without systematic patient-reported assessment, oral symptoms and their functional consequences may remain underrecognised in routine oncology care [4, 11].

Patient-reported outcome measures can facilitate the systematic assessment of treatment-related symptoms and their consequences. However, standardised symptom and quality-of-life scores may not fully capture how patients perceive changes in their dental and gingival health during cancer treatment, how these changes affect the amount and type of food consumed, or which counselling services and self-management strategies they use. Evidence integrating these interrelated aspects remains limited, particularly in heterogeneous populations receiving different forms of oncological treatment. The added value of an integrated patient-reported approach lies in examining not only the frequency of oral complaints, but also perceived changes from the pre-treatment situation, their functional consequences for eating and quality of life, and the supportive-care and self-management strategies used by patients within the same survey.

The aim of this study was to characterise patient-reported oral complaints experienced in relation to cancer treatment, assess their associations with eating-related consequences and perceived quality-of-life impairment, and describe the support, counselling, and self-management strategies reported by participants.

## Methods

### Study design and participants

This cross-sectional, questionnaire-based study was conducted in Germany among adults with a diagnosis of cancer who were receiving or had previously received oncological treatment. Participants were eligible if they were aged 18 years or older, had received chemotherapy, radiotherapy, immunotherapy, or another oncological treatment modality, either alone or in combination. The questionnaire was available in German only. Participants were therefore required to be able to understand and complete the German-language questionnaire. However, German-language proficiency was not formally assessed using a standardised measure or predefined threshold. Participants were recruited through five outpatient oncology centres in Germany and via an online questionnaire distributed through the Haus der Krebs-Selbsthilfe – Bundesverband e. V. (HKSH-BV). At the outpatient oncology centres, adult patients with a diagnosis of cancer who were receiving or had previously received oncological treatment were offered the paper questionnaire and were asked to return the completed questionnaire anonymously in a closed box. For the online recruitment pathway, the self-help organisation was contacted by email and subsequently distributed the survey link among its members. The number of potentially eligible individuals approached was not systematically recorded. Therefore, a formal response rate could not be calculated. Current treatment status and the time elapsed since treatment completion were not systematically recorded at the individual participant level. No formal power-based sample size calculation was performed because the study was designed as an exploratory cross-sectional survey. The study protocol anticipated the recruitment of at least 100 participants. Recruitment continued until this anticipated sample size had been substantially exceeded and was subsequently closed, resulting in a final sample of 222 participants.

### Questionnaire

A study-specific, self-administered questionnaire comprising 31 items was developed by the first author and subsequently reviewed and revised in close consultation with co-author Jutta Hübner. The questionnaire was designed to capture participant and treatment characteristics, patient-reported dental and gingival complaints in relation to cancer treatment, xerostomia, taste alterations, eating-related consequences, perceived quality-of-life impairment, support and counselling, communication with healthcare professionals, and self-management strategies. Treatment-related information included broad treatment modality and treatment duration. Tumour type or site, specific systemic treatment regimens or agents, and detailed radiotherapy characteristics such as treatment field and dose were not collected.

A study-specific instrument was chosen because the aims of the survey extended beyond the assessment of oral health-related quality of life alone. Established instruments such as the OHIP-14 [12, 13] do not comprehensively capture the combination of treatment-associated oral complaints, eating-related consequences, counselling, and self-management strategies addressed in the present study. The study was also intended to generate practical patient-reported information that could subsequently inform patient-facing educational material, including advice on eating-related difficulties and supportive self-management strategies. These objectives required more detailed information on specific symptoms and patient experiences than an oral-health-related quality-of-life instrument alone would provide.

The selection of content domains was informed by established cancer- and oral-health-related quality-of-life instruments, including the EORTC QLQ-C30, EORTC QLQ-H&N35, and EORTC QLQ-OH15 [5, 14, 15]. These instruments served as conceptual references at the domain level, while the questionnaire itself was developed specifically for the present study. The questionnaire domains and items were selected to reflect the aims of the study and the patient-reported experiences of interest. However, no formal structured assessment of content validity using predefined rating procedures or quantitative content-validity measures was performed.

Before data collection, the questionnaire was informally pretested with laypersons to assess the comprehensibility of the wording and response options. No formal cognitive testing or pilot study in the target oncology population was performed, and the questionnaire did not undergo formal psychometric validation.

Most items provided predefined response options, including binary, single-choice, multiple-choice, and ordinal response formats. Selected items included optional free-text fields, and routing instructions allowed participants to skip follow-up questions that were not applicable. The principal symptom-related items referred to experiences and changes during cancer treatment. Participants who had already completed treatment therefore reported retrospectively on the respective treatment period. For participants who had received multiple treatment modalities or previous treatment episodes, no additional instruction specified which treatment episode should serve as the reference period. Selected items, such as the question concerning the fit of dental prostheses, referred to experiences during or after treatment.

All oral complaints and symptoms were patient-reported and were not clinically verified. No additional clinical definition of “dental and gingival complaints” was provided beyond the wording and response options in the questionnaire. Examples and categories included gingival inflammation or pain, gingival bleeding, dry mouth, pain during chewing, tooth sensitivity, oral wounds or ulcers, and caries or other dental problems. The term therefore refers to patient-reported complaints rather than clinically defined or verified oral conditions. Xerostomia was assessed as subjective dry mouth. No objective measurement of salivary flow or salivary gland function was performed. Perceived quality-of-life impairment was assessed using a single study-specific item and did not represent a validated multidimensional quality-of-life measure. Reduced food intake referred to a patient-reported reduction in the amount of food consumed and was not intended as a quantitative measure of energy or nutrient intake or nutritional status. Oral-health support or counselling was not defined using a standardised framework beyond the wording of the respective questionnaire item. Its specific content, intensity, timing, and duration were not systematically recorded.

### Data collection and ethical considerations

Data were collected between January and June 2025 using paper-based questionnaires and an online version administered via SoSci Survey. Participation was voluntary, and all data were collected anonymously. Return of a completed paper questionnaire or electronic submission of the online questionnaire was considered consent to participate. All participants received the same standardised questionnaire. The questionnaire was designed for self-administration. However, whether it was completed without assistance was not formally recorded.

The study was approved by the Ethics Committee of the Faculty of Medicine at Friedrich Schiller University Jena (2024-3628-Bef). The study was conducted in accordance with the relevant guidelines and regulations, including the Declaration of Helsinki.

### Statistical analysis

Data were analysed using IBM SPSS Statistics, version 31. Missing data were not imputed. Analyses were based on valid responses for the variables included in each analysis, and denominators therefore varied across outcomes. For conditional follow-up items, only participants who were eligible to answer the respective question and provided a valid response were included in the denominator.

Categorical variables are presented as absolute and relative frequencies. Continuous variables are summarised using the mean and standard deviation, median and interquartile range, and range. For selected key prevalence estimates, 95% confidence intervals (CIs) were calculated using the Wilson score method without continuity correction. These calculations were based on respondents with valid responses to the respective items, excluding missing and non-applicable responses.

Associations between categorical variables were examined using cross-tabulations and Pearson’s chi-square tests, with Cramér’s V reported as a measure of effect size. For selected 2 × 2 associations, unadjusted odds ratios (ORs) with 95% confidence intervals were calculated from the corresponding contingency tables using the logarithmic Wald method. Associations involving ordered categories were assessed using linear-by-linear association tests.

Additional exploratory analyses examined gender differences in xerostomia, reduced food intake, dietary impact, and difficulties consuming specific food categories. Dietary impact was dichotomised into no impact versus at least mild impact because of sparse observations in the higher-severity categories. Associations were assessed using Pearson’s chi-square tests, with Cramer’s V reported as a measure of effect size where appropriate. Two-sided Fisher’s exact tests were used for food-category comparisons involving smaller subgroups and sparse responses.

As exploratory sensitivity analyses, multivariable binary logistic regression models were fitted to examine whether the associations between new, additional, or worsening dental and gingival complaints and three patient-reported outcomes (reduced food intake, perceived quality-of-life, and receipt of oral-health support or counselling) persisted after adjustment for age and gender. Age was modelled per 10-year increase. Unadjusted and adjusted odds ratios with 95% confidence intervals were calculated using identical complete-case samples for each outcome. These models were intended as limited sensitivity analyses rather than as comprehensive multivariable adjustment because several clinically relevant potential confounders, including tumour characteristics, detailed treatment exposure, treatment status, and comorbidities, were unavailable.

All tests were two-sided, and *p* < 0.05 was considered statistically significant. All inferential analyses were exploratory and intended to generate hypotheses rather than provide confirmatory evidence. No adjustment for multiple testing was performed because no formal hypothesis-testing hierarchy or multiplicity framework had been prespecified. Consequently, p-values were interpreted cautiously alongside effect estimates and 95% confidence intervals.

## Results

### Study population

A total of 222 participants with current or previous oncological treatment took part in the survey. The mean age was 64.2 ± 12.1 years, and 63.1% of participants were female. Of the 222 participants, 195 (87.8%) were recruited through the five outpatient oncology centres, while 27 (12.2%) completed the online questionnaire distributed through the patient self-help organisation. Further participant and treatment characteristics are presented in Table 1.

**Table 1:** Characteristics of the study population

Characteristic	Category / parameter	Result, n (%) or value
Total sample	Participants	222
Age, years (*n* = 216)	Mean ± SD	64.2 ± 12.1
	Median [IQR]	66.0 [58.3–73.0]
	Range	27–89
Gender (*n* = 222)	Female	140 (63.1%)
	Male	82 (36.9%)
	Diverse	0 (0.0%)
Treatment modality (*n* = 207)*	Chemotherapy	165 (79.7%)
	Immunotherapy	56 (27.1%)
	Radiotherapy	15 (7.2%)
	Other treatment modality	20 (9.7%)
Treatment duration (*n* = 206)	< 3 months	34 (16.5%)
	3–6 months	68 (33.0%)
	> 6 months	104 (50.5%)

* Multiple treatment modalities could be reported; therefore, percentages do not add up to 100%. All other percentages are based on valid responses for the respective variable

### Dental and gingival complaints before and during cancer treatment

Pre-existing dental or gingival complaints before cancer treatment were reported by 28.4% (*n* = 61) of 215 respondents. Of the 59 participants who specified the type of complaint, tooth sensitivity was reported most frequently (35.6%, *n* = 21), followed by gingival bleeding (32.2%, *n* = 19), gingival inflammation or pain (30.5%, *n* = 18), caries (30.5%, *n* = 18), and other dental problems (10.2%, *n* = 6).

Fixed dental prostheses or restorations, such as crowns, bridges, or implant-supported restorations, were reported by 45.8% (*n* = 98) of 214 respondents, while 25.7% (*n* = 55) reported removable dental prostheses. The subsequent question on fit and usability during or after cancer treatment applied to participants with either fixed or removable dental prostheses. Of the 153 respondents reporting either type of prosthetic restoration, 142 provided valid information on fit and usability. Most reported no change (83.8%, *n* = 119), whereas 11.3% (*n* = 16) stated that their prostheses no longer fitted properly and 4.9% (*n* = 7) reported that they were unable to wear them.

During cancer treatment, 41.9% (83/198; 95% CI 35.3–48.9%) of respondents reported newly developed, additional, or worsening dental and gingival complaints. Complaints developed for the first time in 21.2% (*n* = 42), while 15.2% (*n* = 30) reported additional new complaints and 5.6% (*n* = 11) reported worsening of pre-existing complaints. By contrast, 57.1% (*n* = 113) reported no change, and 1.0% (*n* = 2) reported an improvement in pre-existing complaints.

The time of onset was reported by 73 respondents. Complaints most frequently began after more than 3 months of treatment (34.2%, *n* = 25), followed by onset within the first month (30.1%, *n* = 22) and after 1–3 months (24.7%, *n* = 18). A further 11.0% (*n* = 8) provided another time specification.

Participants with newly developed, additional, or worsening complaints reported perceived quality-of-life impairment attributed to dental or gingival complaints more frequently than those reporting no change or improvement. Perceived quality-of-life impairment was reported by 50.7% (37/73) of participants with such complaints, compared with 7.1% (7/98) of those reporting no change or improvement, corresponding to an unadjusted OR of 13.36 (95% CI 5.46–32.71) (χ² = 41.51, *p* < 0.001, Cramér’s V = 0.49).

Participants with newly developed, additional, or worsening complaints also reported reduced food intake more frequently than those reporting no change or improvement. Reduced food intake was reported by 51.3% (41/80) and 12.0% (13/108), corresponding to an unadjusted OR of 7.68 (95% CI 3.71–15.89) (χ² = 34.52, *p* < 0.001, Cramér’s V = 0.43).

### Xerostomia and eating-related outcomes

**Fig. 1:**
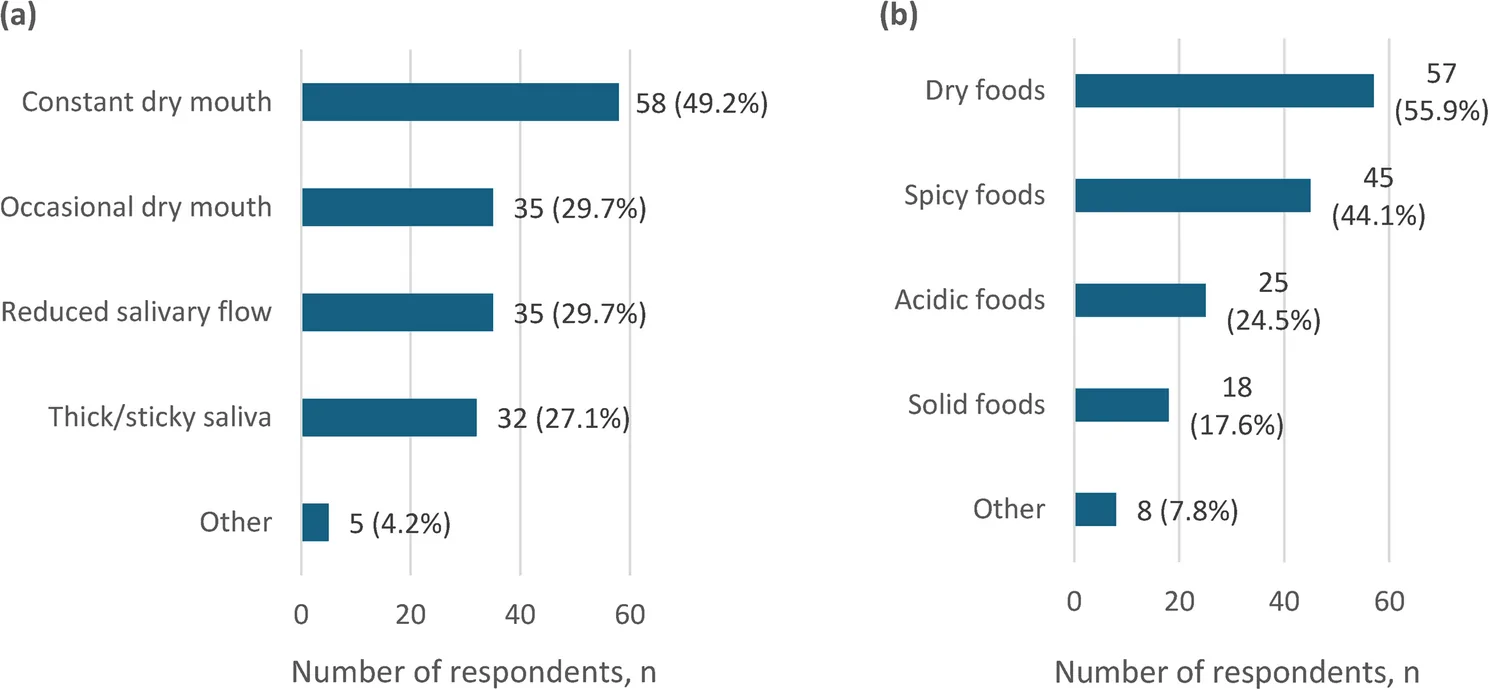
Manifestations of xerostomia and associated eating difficulties during cancer treatment. **a** Reported manifestations of xerostomia (*n* = 118). **b** Foods reported as difficult to consume because of xerostomia (*n* = 102). Multiple responses were possible; percentages are based on respondents with valid information for the respective item

During cancer treatment, xerostomia was reported by 56.6% (120/212; 95% CI 49.9–63.1%) of respondents. Of the 118 participants who characterised their symptoms, constant dry mouth, including at night, was the most frequently reported manifestation (49.2%, *n* = 58). The distribution of other reported manifestations are shown in Fig. 1a.

The perceived effect of xerostomia on eating was rated by 117 respondents, of whom 48.7% (*n* = 57) reported at least slight difficulties. These difficulties were classified as slight by 26.5% (*n* = 31), moderate by 12.0% (*n* = 14), and severe by 10.3% (*n* = 12), whereas 51.3% (*n* = 60) reported no difficulty. A self-reported reduction in the amount of food consumed because of xerostomia was additionally reported by 29.6% (*n* = 34) of 115 respondents.

Of the 102 respondents who identified foods that were difficult to consume in the context of xerostomia, dry foods were reported most frequently (55.9%, *n* = 57). The distribution of reported food categories is shown in Fig. 1b. Multiple responses were possible. Measures to alleviate xerostomia were reported by 63.4% (*n* = 71) of 112 respondents.

Among the 115 respondents with xerostomia who provided information on both eating difficulties and food intake, the proportion reporting reduced food intake increased with the severity of xerostomia-related eating difficulties. Reduced food intake was reported by 5.1% (*n* = 3) of those with no difficulty, 40.0% (*n* = 12) of those with slight difficulty, 57.1% (*n* = 8) of those with moderate difficulty, and 91.7% (*n* = 11) of those with severe difficulty. This ordered association was statistically significant (linear-by-linear association, χ² = 44.81, exact two-sided *p* < 0.001, Cramér’s V = 0.63).

### Dietary outcomes, taste alterations, and perceived quality-of-life impairment

At least a mild dietary impact attributed to dental or gingival complaints was reported by 37.8% (*n* = 76) of 201 respondents. Detailed distributions of dietary-impact severity and food categories reported as difficult to consume are presented in Table 2. Taste alterations compared with the pre-treatment period were reported by 68.0% (138/203; 95% CI 61.3–74.0%) of respondents.

**Table 2:** Oral symptoms and associated consequences

Domain	Outcome or category	% (*n*/*N*)
Dietary impact of dental or gingival complaints	No impact	62.2% (125/201)
	Mild impact	20.9% (42/201)
	Moderate impact	10.4% (21/201)
	Severe impact	5.5% (11/201)
	Very severe impact	1.0% (2/201)
Foods difficult to consume because of dental or gingival complaints*†	Hard or solid foods	66.2% (47/71)
	Spicy foods	57.7% (41/71)
	Acidic foods	36.6% (26/71)
	Hot foods or beverages	29.6% (21/71)
	Sugary foods	19.7% (14/71)
	Cold foods or beverages	18.3% (13/71)
	Other foods	5.6% (4/71)
Taste alterations during cancer treatment	Pronounced alteration	27.6% (56/203)
	Noticeable alteration	33.0% (67/203)
	Slight alteration	7.4% (15/203)
	No alteration	32.0% (65/203)
Food-related consequences and adaptations	Reduced food intake attributed to dental or gingival complaints	27.9% (57/204)
	Avoidance of foods because of oral pain or discomfort	27.6% (54/196)
	Specific dietary adaptations	19.4% (33/170)
Preferred foods in the presence of dental or gingival complaints*	Soft food	40.7% (77/189)
	Liquid food	13.8% (26/189)
	Mild food	33.9% (64/189)
	Cold food	6.9% (13/189)
	Warm food	18.0% (34/189)
	Other food	3.7% (7/189)
	No dental or gingival complaints	40.7% (77/189)
Perceived quality-of-life impairment	Impairment attributed to dental or gingival complaints	25.0% (46/184)

* Multiple responses were possible† Assessed among respondents who reported at least a mild dietary impact and selected at least one response option in the corresponding follow-up item

Reduced food intake attributed to dental or gingival complaints was reported by 27.9% (57/204; 95% CI 22.2–34.5%) of respondents. This outcome referred to a reduction in the amount of food consumed and was distinct from the broader dietary-impact item reported above, which captured any degree of perceived influence on diet. Further food-related consequences and adaptations are summarised in Table 2. Perceived quality-of-life impairment attributed to dental or gingival complaints during cancer treatment, as assessed by the single study-specific item, was reported by 25.0% (46/184; 95% CI 19.3–31.7%) of respondents (Table 2).

In exploratory gender comparisons, women numerically reported xerostomia more frequently than men (61.0% [83/136] vs. 48.7% [37/76]; *p* = 0.082). Women also numerically reported a subjective reduction in food intake that they attributed to dental or gingival complaints (32.6% [42/129] vs. 20.0% [15/75]; *p* = 0.054). The prevalence of at least mild dietary impact was similar between women and men (37.2% [48/129] vs. 38.9% [28/72]; *p* = 0.814). No statistically significant gender differences were identified for individual food categories reported as difficult to consume in relation to xerostomia (all *p* ≥ 0.180) or dental or gingival complaints (all *p* ≥ 0.127). These analyses were exploratory and were not adjusted for multiple testing.

### Support, counselling, self-management, and communication

Support or counselling regarding dental and oral health during cancer treatment was reported by 43.1% (87/202; 95% CI 36.4–50.0%) of respondents. Of the 74 respondents who rated the support received, 85.1% (*n* = 63) considered it helpful or very helpful. Detailed information on the frequency, sources, and perceived helpfulness of support is presented in Table 3.

**Table 3:** Support and management of oral complaints

Domain	Outcome or category	Result, % (*n*/*N*)
Support and counselling	Support received regularly	12.9% (26/202)
	Support received occasionally	30.2% (61/202)
	No support received	56.9% (115/202)
Source of support*†	Dentist	55.0% (44/80)
	Oncologist	52.5% (42/80)
	Nursing staff	32.5% (26/80)
	Oral and maxillofacial surgeon	5.0% (4/80)
	Other source	11.3% (9/80)
Helpfulness of support†	Very helpful	25.7% (19/74)
	Helpful	59.5% (44/74)
	Slightly helpful	14.9% (11/74)
	Not helpful	0.0% (0/74)
Self-management	Any measure or strategy tried	52.4% (100/191)
Type of measure or strategy*‡	Special mouthwashes	63.3% (62/98)
	Toothpastes for sensitive teeth	60.2% (59/98)
	Dietary adaptations	40.8% (40/98)
	Regular professional dental cleaning	26.5% (26/98)
	Saliva substitutes or sprays	15.3% (15/98)
	Other measures	14.3% (14/98)
Communication with healthcare professionals	Very satisfied	28.6% (50/175)
	Satisfied	31.4% (55/175)
	Neutral	35.4% (62/175)
	Dissatisfied	4.0% (7/175)
	Very dissatisfied	0.6% (1/175)

* Multiple responses were possible

† Sources and helpfulness of support were assessed among respondents who reported receiving support or counselling

‡ Types of measures were assessed among respondents who reported having tried at least one measure or strategy. Percentages are based on respondents eligible for and answering the respective item

Measures or strategies to alleviate dental or gingival complaints had been tried by 52.4% (*n* = 100) of 191 respondents. The types of self-management strategies and ratings of communication with healthcare professionals are summarised in Table 3. The questionnaire did not distinguish between specific mouthwash products or active ingredients and did not record whether the reported measures had been recommended by healthcare professionals or selected independently by participants.

Participants with newly developed, additional, or worsening dental and gingival complaints reported receiving support or counselling more frequently than those with no change or improvement (60.3% [47/78] vs. 31.5% [34/108]; unadjusted OR 3.30, 95% CI 1.80–6.07; χ² [1, *N* = 186] = 15.25, *p* < 0.001, Cramér’s V = 0.29). Nevertheless, 39.7% (*n* = 31) of the 78 participants with newly developed, additional, or worsening complaints reported receiving no support or counselling.

In exploratory sensitivity analyses adjusted for age and gender, new, additional, or worsening dental and gingival complaints remained associated with reduced food intake (adjusted OR 7.85, 95% CI 3.70-16.62; *n* = 185), perceived quality-of-life impairment (adjusted OR 15.25, 95% CI 5.81–40.06; *n* = 168), and receipt of oral-health support or counselling (adjusted OR 3.23, 95% CI 1.72–6.07; *n* = 183). Adjustment for age and gender resulted in only minor changes in the estimates compared with the corresponding complete-case unadjusted models.

## Discussion

This cross-sectional survey provides an integrated patient-reported perspective on oral complaints, their functional consequences, and supportive care during cancer treatment. More than two in five participants reported new, additional, or worsening dental or gingival complaints, and these complaints were associated with reduced food intake and perceived quality-of-life impairment. These associations changed only minimally in exploratory sensitivity analyses adjusting for age and gender, although the limited covariate information precludes conclusions regarding independent effects. Patient-reported xerostomia and taste alterations were also common, and reduced food intake became more frequent with increasing severity of xerostomia-related eating difficulties. Fewer than half of respondents reported receiving oral-health support or counselling, whereas most of those who received and rated such support considered it helpful or very helpful. These findings show that patient-reported oral complaints are relevant not only as symptoms but also because of their associations with eating and perceived quality-of-life impairment.

The frequency of patient-reported changes in dental and gingival health is consistent with the broader evidence that oral complications are common across oncological populations and treatment settings. Oral manifestations have been reported in approximately 60% of adults receiving chemotherapy in a clinical cross-sectional study [16], while a six-month prospective study among patients receiving chemotherapy for solid tumours found a cumulative incidence of 43.8% for oral mucositis of grade 2 or higher [17]. Prospective assessments during radiotherapy or chemoradiotherapy for head and neck cancer have likewise demonstrated increasing oral symptom burden during treatment, with some problems persisting after treatment completion [18]. Previous patient-reported research has also described a broad spectrum of oral problems among patients receiving chemotherapy for solid tumours [4]. Direct comparison of prevalence estimates is limited, however, by differences in cancer type, treatment modality, observation period, symptom definition, and assessment method. Rather than focusing on clinically assessed mucosal toxicity or a specific treatment group, the present study captured patient-reported changes in dental and gingival health relative to the pre-treatment situation across a heterogeneous oncological population. The reported onset of complaints at different stages of treatment suggests that these problems may not be confined to a single treatment phase. However, because the study was cross-sectional and participants who had completed treatment reported retrospectively, individual symptom trajectories, persistence, and the timing of symptom resolution cannot be established. These observations do not establish the optimal timing or effectiveness of repeated oral-health assessment. Rather, they provide a rationale for prospectively evaluating whether repeated assessment at different stages of cancer treatment can improve the recognition and management of oral complaints.

The functional relevance of these complaints is reflected in their relationship with eating and perceived quality-of-life impairment. Participants with new, additional, or worsening dental or gingival complaints reported reduced food intake substantially more often than those reporting no change or improvement. This association remained similar after exploratory adjustment for age and gender. However, the analysis could not account for important clinical factors or other treatment-related symptoms that may also influence food intake. More broadly, 37.8% of respondents reported at least some dietary impact of dental or gingival complaints, whereas 27.9% specifically reported a reduction in the amount of food consumed, reflecting two distinct patient-reported outcomes. Difficulties consuming hard or solid, spicy, and acidic foods, together with the frequent preference for soft and mild foods, further illustrate perceived changes in both the amount and type of food consumed. This is consistent with observational studies linking oral symptoms to reduced oral intake and nutritional risk. In patients with head and neck cancer, decreasing oral intake during radiotherapy has been associated with patient-reported factors such as loss of taste, reduced interest in food, and loss of hunger [19]. In a heterogeneous oncological population, self-reported oral health complaints were also associated with nutritional risk and malnutrition [20]. The present study did not assess actual energy or nutrient intake, body-weight changes, or nutritional status; self-reported reductions in food intake should therefore not be interpreted as evidence of clinically diagnosed malnutrition. Nevertheless, oral complaints that interfere with eating may provide an additional reason to consider validated nutritional screening. The current German S3 guideline on clinical nutrition in oncology recommends early and repeated screening for malnutrition and, following an abnormal result, comprehensive nutritional assessment and individualised counselling by qualified professionals [21]. The observed association with perceived quality-of-life impairment further underlines that the relevance of dental and gingival complaints extends beyond local discomfort, although this outcome was assessed using a single study-specific item and should therefore be interpreted as perceived complaint-related impairment rather than as a comprehensive multidimensional assessment of quality of life.

Xerostomia and taste alterations were among the most frequently reported symptoms. The most notable xerostomia-related finding was the pronounced increase in reduced food intake across increasing levels of eating difficulty. This association suggests that the perceived functional burden of xerostomia may be particularly relevant for supportive-care needs, beyond the presence of subjective dry mouth alone. Saliva contributes to lubrication, bolus formation, swallowing, taste perception, oral clearance, and the protection of oral tissues [22]. Although xerostomia is a subjective symptom and salivary gland hypofunction was not objectively assessed in the present study, reduced salivary function can interfere with eating and increase susceptibility to dental caries, candidiasis, mucosal irritation, and periodontal complications [23]. The reported difficulties with dry, spicy, acidic, and solid foods are consistent with these functional consequences. Taste alterations may further affect appetite, food selection, and dietary intake and are well-recognised during chemotherapy. Recent studies have associated increasing severity of taste alterations with nutritional risk, difficulties meeting energy requirements, weight loss, and poorer quality of life [24, 25]. Although chemotherapy was the most frequently reported treatment modality in the present cohort, the observed prevalence of taste alterations cannot be attributed to a specific treatment modality because treatment groups overlapped and detailed treatment regimens were not recorded. Moreover, taste alterations were not analysed separately in relation to food intake. Their independent contribution therefore cannot be distinguished from that of xerostomia or other concurrent oral complaints. The questionnaire also did not systematically assess the duration or persistence of taste alterations after treatment. Therefore, the present data cannot distinguish transient from longer-term changes.

Oral-health support or counselling was reported by fewer than half of respondents. Although support or counselling was reported more frequently by participants with new, additional, or worsening complaints, almost two in five participants in this group remained without such assistance. At the same time, 85.1% of those who received and rated support considered it helpful or very helpful, indicating high perceived helpfulness among recipients while suggesting a potential gap in the reach of such support. However, because the content, intensity, and timing of support or counselling were not systematically recorded, the present study cannot assess the quality or adequacy of the support received. This potential gap in reach is consistent with patient-reported evidence indicating that oral and dental needs may remain insufficiently recognised in oncology care unless they are specifically assessed [4, 11]. More than half of the participants had also tried measures to alleviate their complaints, most commonly special mouthwashes, toothpastes for sensitive teeth, and dietary adaptations. These findings demonstrate that patients actively manage oral symptoms in everyday life, but they do not establish whether the measures used were appropriate or effective. Furthermore, the questionnaire did not distinguish between specific mouthwash products or active ingredients and did not record whether self-management measures were professionally recommended or selected independently by participants. Existing guidance already emphasises structured oral care across different phases of cancer treatment. MASCC/ISOO clinical practice statements describe basic oral care and the management of oral complications in several oncological treatment settings, including care before, during, and after treatment [2, 3, 6]. In Germany, the S3 guideline on supportive care likewise recommends preventive oral-care measures and symptom-oriented management of treatment-related oral complications [7]. The present findings therefore do not necessarily indicate a lack of guidance, but rather suggest that its implementation and reach in routine oncology care warrant further evaluation. A more proactive approach may include assessment of symptoms and their effects on eating together with individualised oral-care advice and clear referral pathways. Depending on the underlying complaint and treatment context, this may involve coordination between oncology professionals, dentists, nursing staff, and nutrition professionals. Future studies should evaluate how such structured oral-health pathways can be implemented across different oncological treatment settings and whether they improve patient-reported and clinical outcomes.

### Strengths and limitations

A strength of this study is its integrated, patient-centred assessment of changes in dental and gingival complaints together with their perceived consequences for food intake and quality of life, as well as counselling and self-management, within the same heterogeneous oncology population.

Several limitations should be considered. First, all outcomes were self-reported and were not verified by clinical dental assessment, objective salivary-flow measurement, taste testing, or objective nutritional parameters. Xerostomia therefore represents subjective dry mouth rather than objectively confirmed salivary gland hypofunction, and reduced food intake should not be interpreted as evidence of nutritional deficiency, malnutrition, or clinically significant weight loss. Perceived quality-of-life impairment was assessed using a single study-specific item rather than a validated multidimensional quality-of-life instrument. Because both oral complaints and their perceived consequences were assessed using the same self-reported questionnaire, the observed associations may also have been affected by common-method bias. Moreover, items assessing reduced food intake and perceived quality-of-life impairment explicitly asked participants to attribute these outcomes to dental or gingival complaints, introducing potential attribution bias and conceptual overlap between the measures. Consequently, the strength of these associations should be interpreted cautiously and cannot be considered evidence of causal relationships.

Second, the study-specific questionnaire had not undergone formal psychometric validation or cognitive testing in the target oncology population. Although the selection of content domains was informed by established cancer- and oral-health-related quality-of-life instruments, no formal structured assessment of content validity was performed. Although it was reviewed within the research team and informally pretested for comprehensibility, the pretest involved laypersons rather than patients with cancer. Therefore, comprehensibility in the target population cannot be assured. German-language proficiency was also not formally assessed using a standardised procedure. In addition, no clinical definition of “dental and gingival complaints” was provided beyond the wording and examples included in the questionnaire, which may have resulted in differences in individual interpretation. These factors limit comparability with validated instruments and may have introduced measurement error.

Third, the survey included participants who were receiving or had previously received oncological treatment, but current treatment status and time since treatment completion were not systematically recorded at the individual level. Participants who had completed treatment therefore reported retrospectively on experiences during treatment, introducing potential recall bias. For participants who had received multiple treatment modalities or previous treatment episodes, no additional instruction specified which treatment episode should serve as the reference period. Attribution of reported complaints to a particular treatment period or modality is therefore not possible. The cross-sectional design additionally precludes assessment of individual symptom trajectories, persistence, or causal relationships. The absence of individual treatment status and time since treatment completion should therefore be considered a major limitation when interpreting the timing and persistence of the reported complaints.

Fourth, tumour type or site, detailed systemic treatment regimens, radiotherapy field and dose, comorbidities, and concomitant medications were not available. Consequently, treatment-specific attribution was not possible, and residual confounding by treatment characteristics, cancer-related factors, gender, and other unmeasured variables cannot be excluded. Other factors not systematically assessed, including mucositis, nausea, swallowing difficulties, appetite changes, and psychological factors, may also have influenced oral symptoms and eating-related outcomes. The exploratory age- and gender-adjusted sensitivity analyses provided only limited adjustment because important clinical confounders remained unavailable. Although the adjusted estimates were similar to the corresponding unadjusted estimates, these analyses should not be interpreted as demonstrating independent effects of dental or gingival complaints.

Fifth, the number of potentially eligible individuals approached was not systematically recorded, and a formal response rate could therefore not be calculated. Recruitment through five outpatient oncology centres and an online patient self-help pathway may have introduced selection and non-response bias and may limit assessment of representativeness. For example, individuals experiencing oral complaints or with a particular interest in oral health may have been more likely to participate, potentially increasing reported symptom frequencies, whereas patients with greater disease or treatment burden may have been less likely to participate. Because information on non-participants was unavailable, the direction and magnitude of these potential biases cannot be determined. Accordingly, the findings should be interpreted primarily in the context of similar outpatient oncology populations, and their transferability to the broader German oncology population or other healthcare settings remains uncertain.

Sixth, oral-health support or counselling was not defined using a standardised framework, and its specific content, intensity, timing, and duration were not systematically recorded. The study therefore cannot determine the quality, appropriateness, or adequacy of the support received or compare different forms of counselling. Similarly, the self-management strategies reported by participants were not clinically evaluated, and the data do not establish their efficacy or appropriateness.

Finally, item-level missingness varied across outcomes, analyses were based on available cases, and no imputation was performed. In addition, multiple statistical comparisons were conducted without formal adjustment for multiplicity because of the exploratory nature of the study, increasing the possibility of type I error. The additional subgroup and sensitivity analyses were likewise exploratory and were not adjusted for multiple testing. Individual statistically significant findings should therefore be interpreted cautiously and considered hypothesis-generating rather than confirmatory.

## Conclusion

In this recruited sample of adults with current or previous oncological treatment, patient-reported dental, gingival, and other oral complaints were common in relation to cancer treatment and were associated with eating-related burden. The marked association between the severity of xerostomia-related eating difficulties and reduced food intake highlights the potential relevance of assessing functional consequences in addition to symptom presence, without establishing a causal relationship. Although counselling was generally perceived as helpful among recipients, fewer than half of respondents reported receiving such support, suggesting a potential gap in its reach. These findings support further prospective evaluation of repeated, function-oriented oral-health assessment, symptom-specific dental care, and appropriate nutritional referral rather than demonstrating the effectiveness of these approaches. Prospective studies using clinical oral assessments and validated patient-reported measures are needed to characterise symptom trajectories and to determine whether structured interdisciplinary care improves patient-reported or clinical outcomes.

## Supplementary Information

Supplementary Material 1.

## Data Availability

The datasets generated and analysed during the current study are available from the corresponding author on reasonable request.
