# Supplementary material for: Patient-reported oral complaints during cancer treatment: Dietary consequences, supportive care, and self-management in a cross-sectional survey
Source: BMC Oral Health. 2026 Nov 3;26:2172. doi: 10.1186/s12903-026-10389-0 (PMC13733013; doi:10.1186/s12903-026-10389-0)
Supplement: Supplementary file 1 [file 12903_2026_10389_MOESM1_ESM.pdf]

Questionnaire on Dental and Gingival Problems During Cancer Treatment and Their Impact on Nutrition

Section 1: General Information

1. Age: ___

2. Sex:

☐ Male
☐ Female
☐ Diverse

3. Type of cancer treatment:

☐ Chemotherapy
☐ Radiotherapy, particularly to the head and neck region
☐ Immunotherapy
☐ Other: _____________

4. Duration of cancer treatment:

☐ < 3 months
☐ 3–6 months
☐ > 6 months

Section 2: Dental Health and Dental Prostheses

5. Did you already have dental or gum problems before starting cancer treatment?

☐ Yes
☐ No (go directly to Question 7)

6. If yes, which dental or gum problems did you have before cancer treatment? Multiple answers are possible.

☐ Gingivitis or gum pain
☐ Bleeding gums
☐ Dry mouth
☐ Pain when chewing
☐ Tooth sensitivity
☐ Sores or ulcers in the mouth
☐ Cavities or other problems with the teeth
☐ Other: _____________

7. Did you have any dental prostheses before cancer treatment?

☐ Yes, fixed dental prostheses, such as crowns, bridges, or implants
☐ Yes, removable dental prostheses, such as partial or complete dentures
☐ No (go directly to Question 9)

8. Does your dental prosthesis still fit properly during or after cancer treatment?

☐ Yes, there has been no change
☐ No, the dental prosthesis no longer fits properly
☐ No, I am currently unable to wear the dental prosthesis at all
If no, what difficulties do you experience? _____________

9. Have your dental and gum problems changed during the course of cancer treatment?

☐ I did not have any dental problems before cancer treatment, but new problems have developed
☐ Yes, existing problems have worsened
☐ Yes, additional new problems have developed
☐ No, there have been no changes (go directly to Question 11)
☐ No, the existing problems have improved (go directly to Question 11)

10. If your existing problems worsened or new problems developed, when did this occur?

☐ Within the first month of treatment
☐ After 1–3 months
☐ After more than 3 months
☐ Other time point: _____________

Section 3: Dry Mouth and Salivary Flow

11. Have you experienced dry mouth during cancer treatment?

☐ Yes
☐ No (go directly to Question 17)

12. If yes, how would you describe your dry mouth? Multiple answers are possible.

☐ Constantly dry mouth, including at night
☐ Dry mouth only occasionally, in the following situations: ___________
☐ A feeling of “thick” or mucus-like saliva
☐ A feeling of reduced salivary flow
☐ Other: _____________

13. Has dry mouth made eating more difficult for you?

☐ Yes, severely
☐ Yes, moderately
☐ Yes, slightly
☐ No

14. Do you feel that dry mouth has reduced the amount of food you eat?

☐ Yes
☐ No

15. Are there any foods that are particularly difficult for you to eat because of dry mouth? Multiple answers are possible.

☐ Dry foods, such as bread or biscuits
☐ Solid foods, such as meat or vegetables
☐ Sour foods, such as citrus fruits
☐ Spicy foods
☐ Other: _____________

16. Have you taken any measures to relieve dry mouth?

☐ Yes
☐ No
If yes, which measures? _____________

Section 4: Dietary Restrictions Due to Dental Problems

17. To what extent have your dental and gum problems affected your diet?

☐ No effect (go directly to Question 19)
☐ Slight restriction
☐ Moderate restriction
☐ Severe restriction
☐ Very severe restriction

18. Which of the following foods are more difficult for you to eat because of your dental problems? Multiple answers are possible.

☐ Hard or solid foods, such as nuts or raw vegetables
☐ Spicy foods
☐ Sour foods, such as citrus fruits
☐ Hot foods or drinks
☐ Cold foods or drinks
☐ Sugary foods
☐ Other: ______________

19. Have foods and drinks tasted different compared with before your treatment?

☐ Yes, very different
☐ Yes, somewhat different
☐ Yes, only slightly different
☐ No

If yes, how would you describe the change, for example saltier or more sour?

---

20. Do you feel that your dental and gum problems have led to reduced food intake?

☐ Yes
☐ No (go directly to Question 22)

21. On a scale from 1 to 5, how strongly do your dental and gum problems affect your daily food intake?

☐ 1 (hardly at all)
☐ 2 (slightly)
☐ 3 (moderately)
☐ 4 (strongly)
☐ 5 (very strongly)

Section 5: Specific Restrictions and Strategies

22. Are there any specific foods that you avoid to prevent pain or discomfort in your mouth?

☐ Yes
☐ No

If yes, which foods? ______________

23. Have you made any specific adjustments to make eating easier, such as puréeing food?

☐ Yes
☐ No

If yes, please describe: ______________

24. What type of food do you prefer when you have dental or gum problems? Multiple answers are possible.

☐ Soft foods, such as purées or soups
☐ Liquid nutrition, such as shakes or smoothies
☐ Mild foods without spicy seasoning
☐ Cold foods
☐ Warm foods
☐ Other: _____________
☐ I do not have any dental or gum problems

Section 6: Impact on Quality of Life

25. Have your dental and gum problems affected your quality of life during treatment?

☐ Yes
☐ No
If yes, in what way? _____________

Section 7: Final Questions and Measures

26. Have you received support or advice regarding dental and oral health during your cancer treatment?

☐ Yes, regularly
☐ Yes, occasionally
☐ No (go directly to Question 28)

If yes, from whom?
☐ Oncologist
☐ Dentist
☐ Oral and maxillofacial surgeon
☐ Nursing staff
☐ Other: _____________

27. How helpful was the support you received in relieving your symptoms?

☐ Very helpful
☐ Helpful
☐ Slightly helpful
☐ Not helpful

28. Have you tried any measures or strategies to relieve dental or gum problems?

☐ Yes
☐ No (go directly to Question 30)

If yes, which measures? Multiple answers are possible.
☐ Special mouthwashes
☐ Toothpastes for sensitive teeth
☐ Saliva substitutes or sprays
☐ Dietary adjustments, such as eating softer foods
☐ Regular professional dental cleanings
☐ Other: _____________

29. Which of these measures did you find particularly effective?

---

30. How satisfied were you with the communication from your treating physicians, such as your oncologist or dentist, regarding possible dental and oral health problems during cancer treatment?

☐ Very satisfied
☐ Satisfied
☐ Neutral
☐ Dissatisfied
☐ Very dissatisfied
If dissatisfied, what could have been improved? _______________

31. Are there any oral-health-related side effects that you have experienced as a result of cancer treatment and about which you would have liked to receive more information beforehand? If so, which ones?

Thank you very much for your participation!
